# Supplementary figures and images for: Chromosome-wide co-fluctuation of stochastic gene expression in mammalian cells
Source: PLoS Genet. 2019 Sep 16;15(9):e1008389. doi: 10.1371/journal.pgen.1008389 (PMC6762216; doi:10.1371/journal.pgen.1008389)

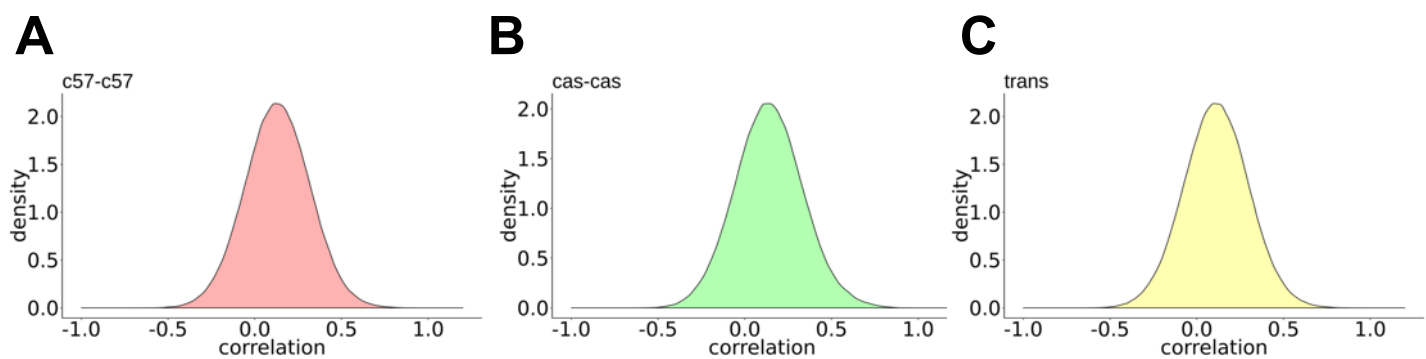

**Figure S1**

Supplement: S1 Fig — (A) The distribution of cis-correlations for alleles on C57BL/6J-derived chromosomes. (B) The distribution of cis-correlations for alleles on CAST/EiJ-derived chromosomes. (C) The distribution of trans-correlation for the C57BL/6J-derived allele at a gene and the CAST/EiJ-derived allele at another gene. (PDF) [file pgen.1008389.s001.pdf]

**A**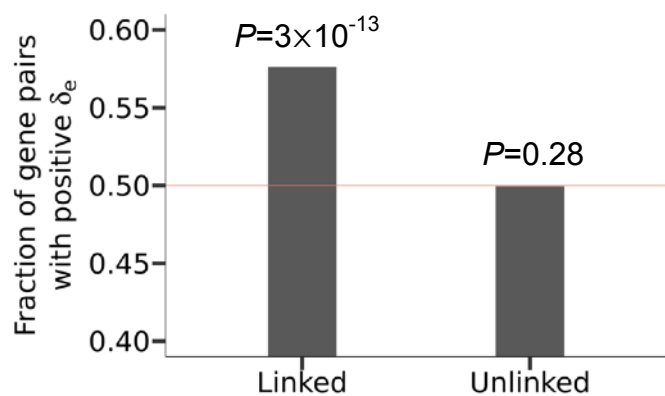**B**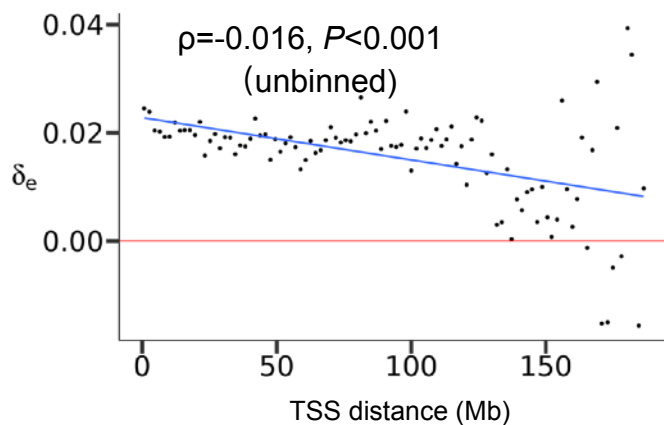**C**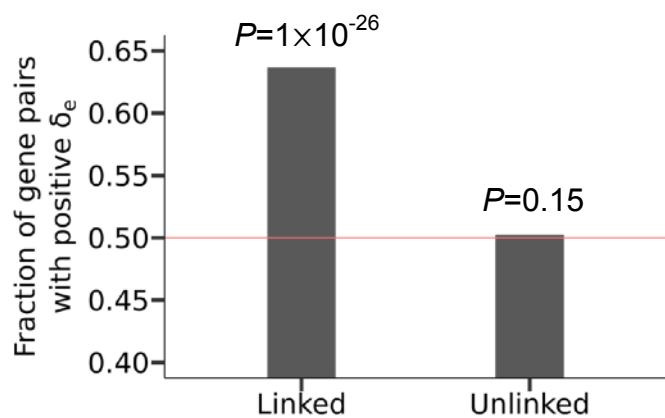**D**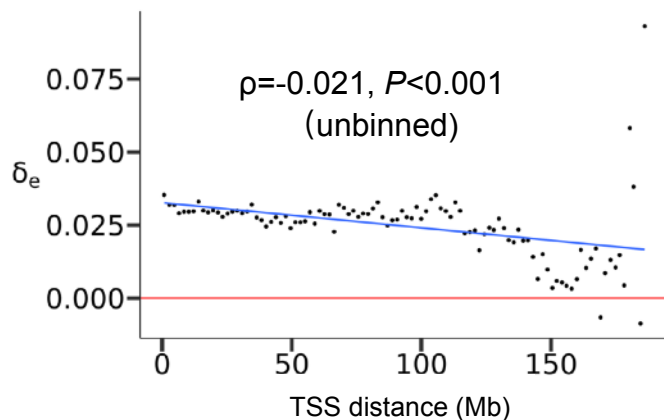**Figure S2**

Supplement: S2 Fig — (A) Fraction of gene pairs with positive δe in clone 6. The red line represents the null expectation under no linkage effect. P-values from binomial tests on independent gene pairs are presented. (B) In clone 6, median δe in a bin decreases as the median genomic distance between linked genes in the bin rises. All bins have the same distance interval. TSS, transcription start site. The red line shows δe = 0. The blue line shows the linear regression of binned data. Spearman's ρ from unbinned data and associated P-value determined by a shuffling test are presented. (C) Fraction of gene pairs with positive δe in non-clonal mouse fibroblast cells. The red line represents the null expectation under no linkage effect. P-values from binomial tests on independent gene pairs are presented. (D) In non-clonal cells, median δe in a bin decreases as the median genomic distance between linked genes in the bin rises. All bins have the same distance interval. The red line shows δe = 0. The blue line shows the linear regression of binned data. Spearman's ρ from unbinned data and associated P-value determined by a shuffling test are presented. (PDF) [file pgen.1008389.s002.pdf]

**A**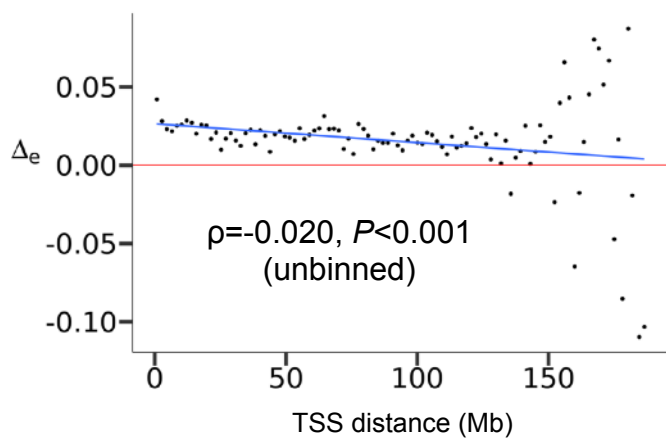**B**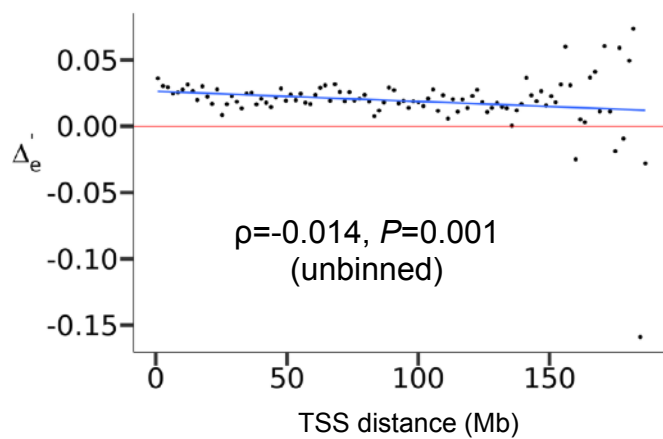**Figure S3**

Supplement: S3 Fig — (A) Median △e in a bin decreases with the median genomic distance between linked genes in the bin. △e for a linked gene pair is the correlation in RNA-seq read number between the two genes minus the median correlation for pairs of unlinked genes. All bins have the same distance interval. TSS, transcription start site. The red line shows △e = 0. The blue line shows the linear regression of binned data. Spearman's ρ of unbinned data and associated P-value determined by a shuffling test are presented. (B) Median △e′ in a bin decreases with the corresponding median genomic distance between linked genes in the bin. △e′ for a linked gene pair is the correlation in expression level measured by RPKM (Reads Per Kilobase per Million mapped reads) between the two genes minus the corresponding median correlation for pairs of unlinked genes. The blue line shows the linear regression of binned data. Spearman's ρ from unbinned data and associated P-value determined by a shuffling test are presented. (PDF) [file pgen.1008389.s003.pdf]

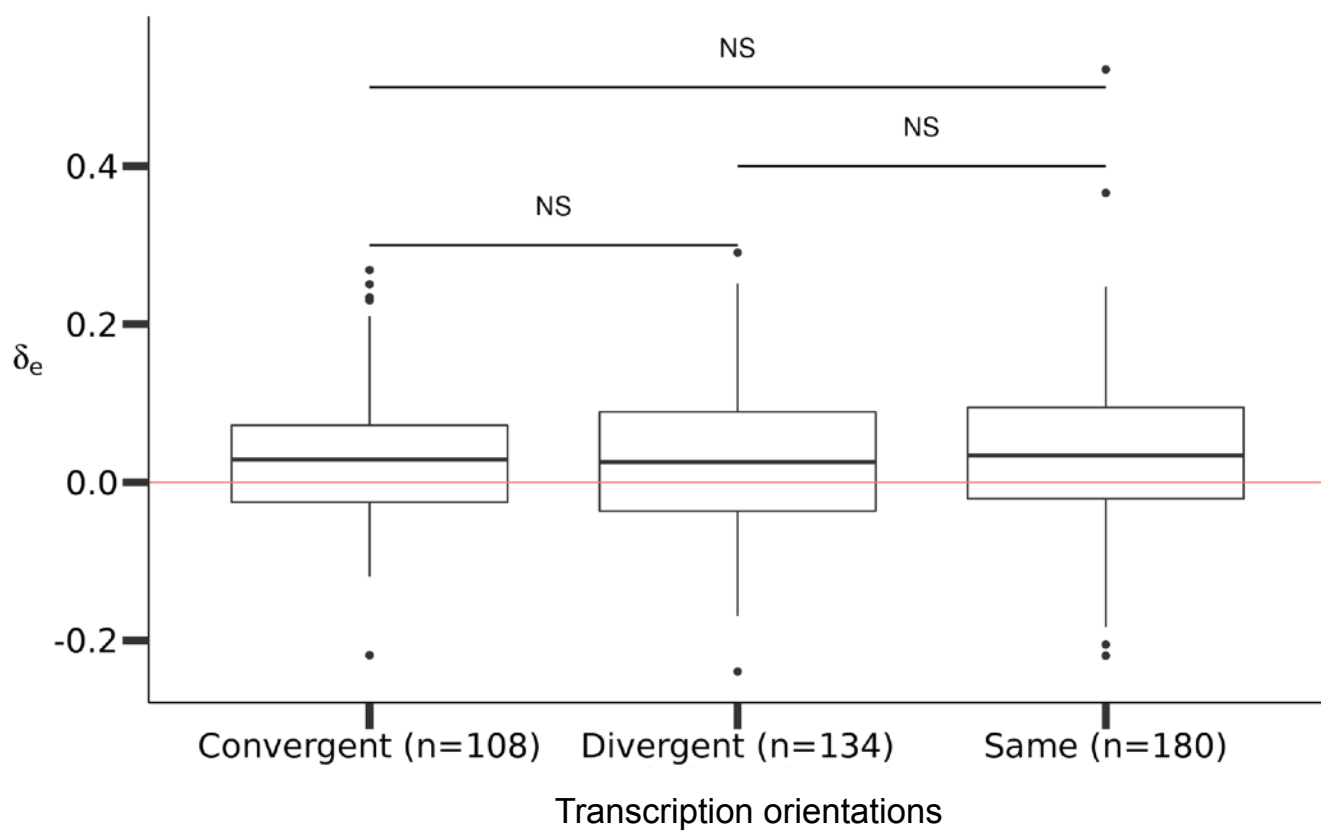

**Figure S4**

Supplement: S4 Fig — The lower and upper edges of a box represent the first (qu1) and third (qu3) quartiles, respectively, the horizontal line inside the box indicates the median (md), the whiskers extend to the most extreme values inside inner fences, md±1.5(qu3-qu1), and the dots represent values outside the inner fences (outliers). The nearest pairs were identified using the coordinates downloaded from Ensembl. After requiring a minimal read number of 10 for each allele, we separate neighboring gene pairs into three categories according to the orientations of their transcription directions. NS, P > 0.05, Wilcoxon rank-sum test. (PDF) [file pgen.1008389.s004.pdf]

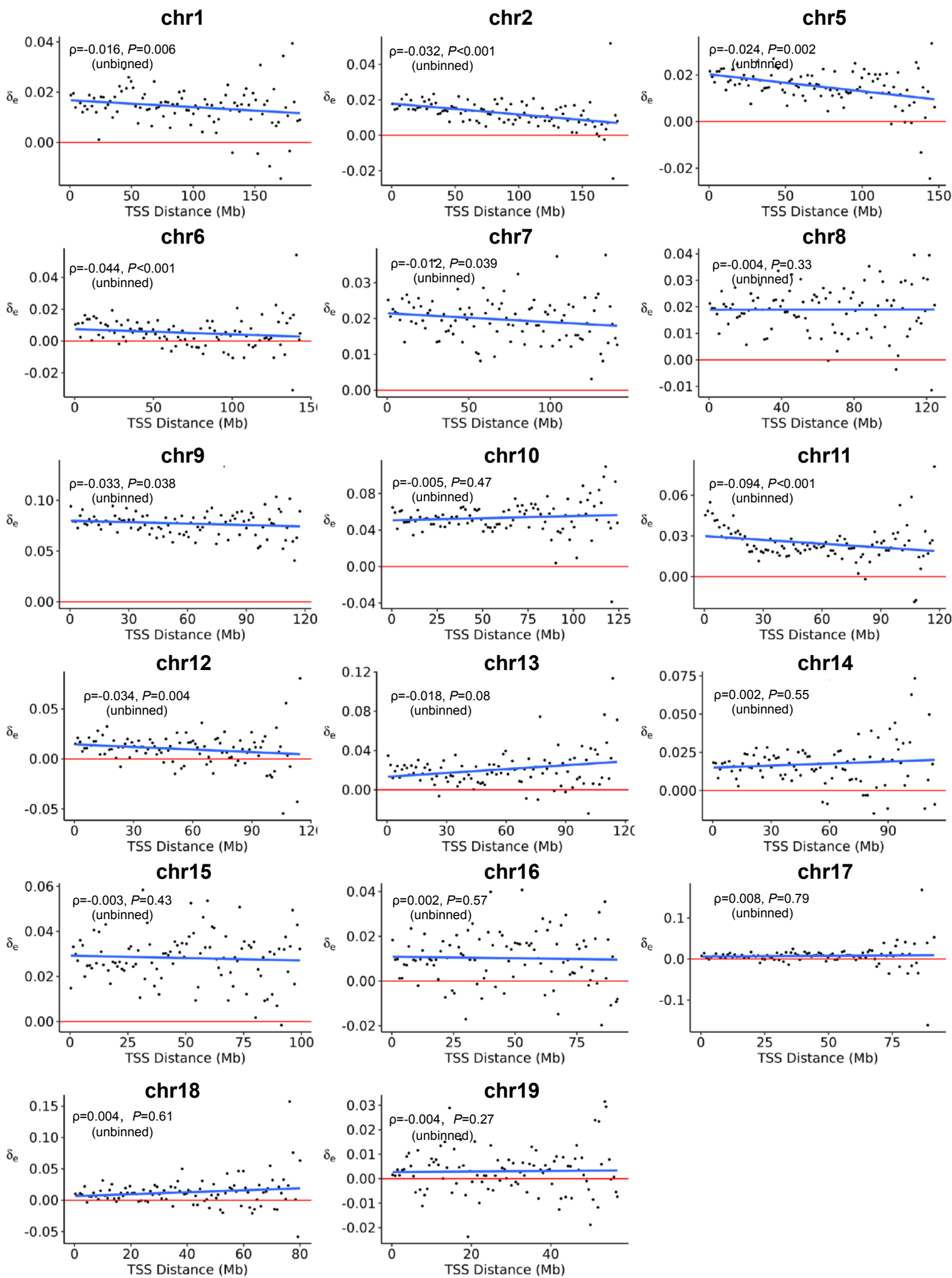

**Figure S5**

Supplement: S5 Fig — Blue lines show linear regressions for binned data. All bins have the same distance intervals, while different chromosomes contain different numbers of bins depending on the chromosome length. Spearman's correlations from unbinned data and associated nominal P-values determined by shuffling tests are presented. Upon multiple testing correction, the correlations remain significant for chromosomes 1, 2, 5, 6, 11, and 12. (PDF) [file pgen.1008389.s005.pdf]

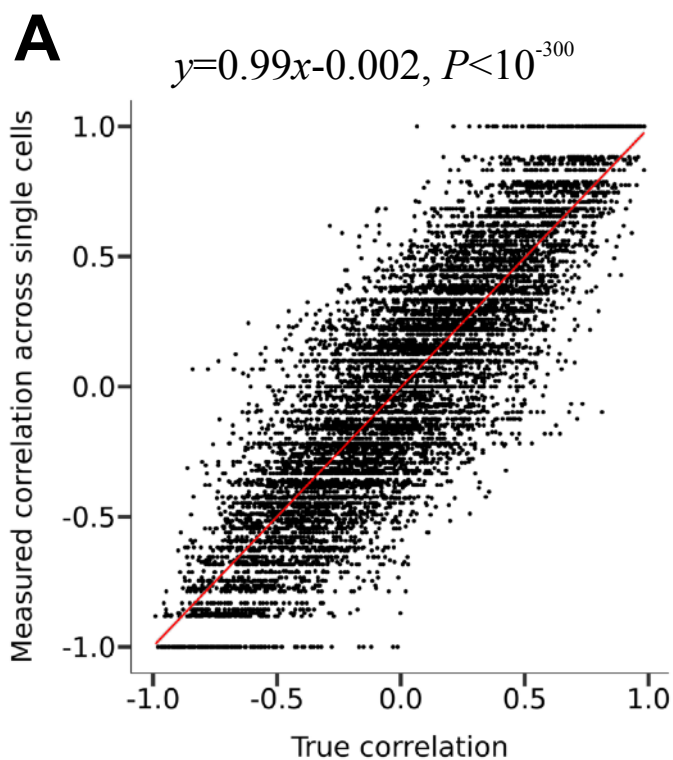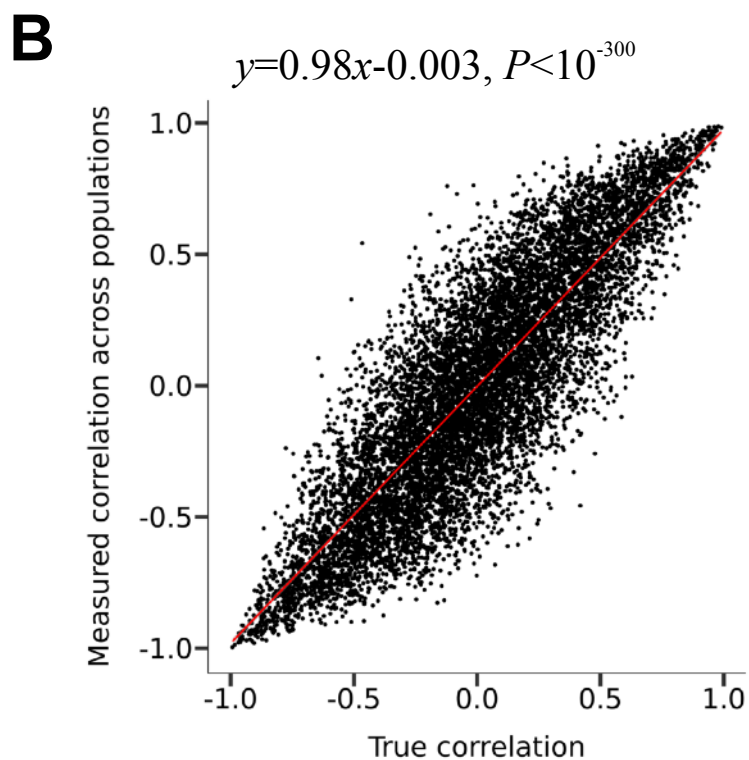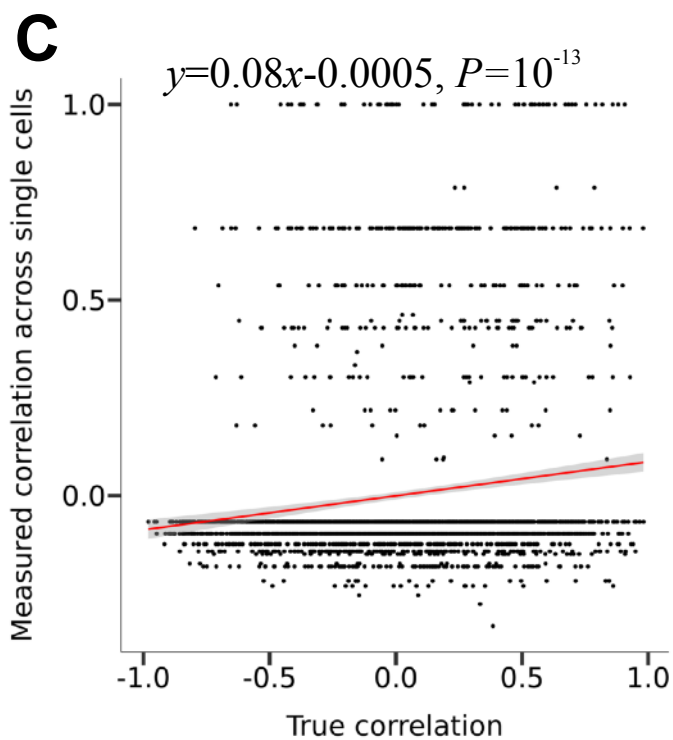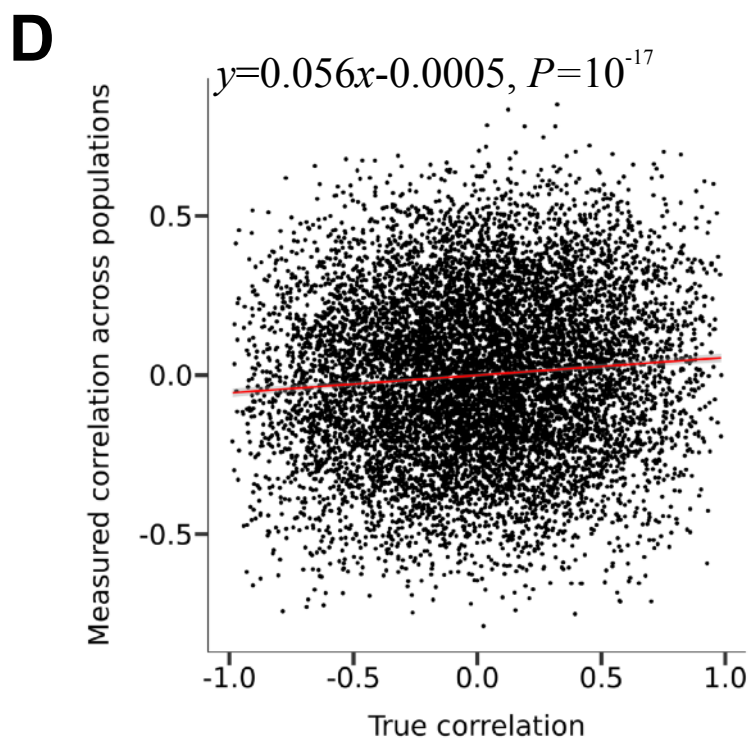

**Figure S6**

Supplement: S6 Fig — (A) The correlations quantified using single-cell-based measurements are close to their corresponding true correlations when the capturing efficiency is 100%. (B) The correlations quantified using cell-population-based measurements are close to the true correlations when the capturing efficiency is 100%. (C) The correlations quantified using single-cell-based measurements tend to be weaker than their corresponding true correlations when the capturing efficiency is 10%. (D) The correlations quantified using cell population-based measurements tend to be weaker than the true correlations when the capturing efficiency is 10%. (PDF) [file pgen.1008389.s006.pdf]

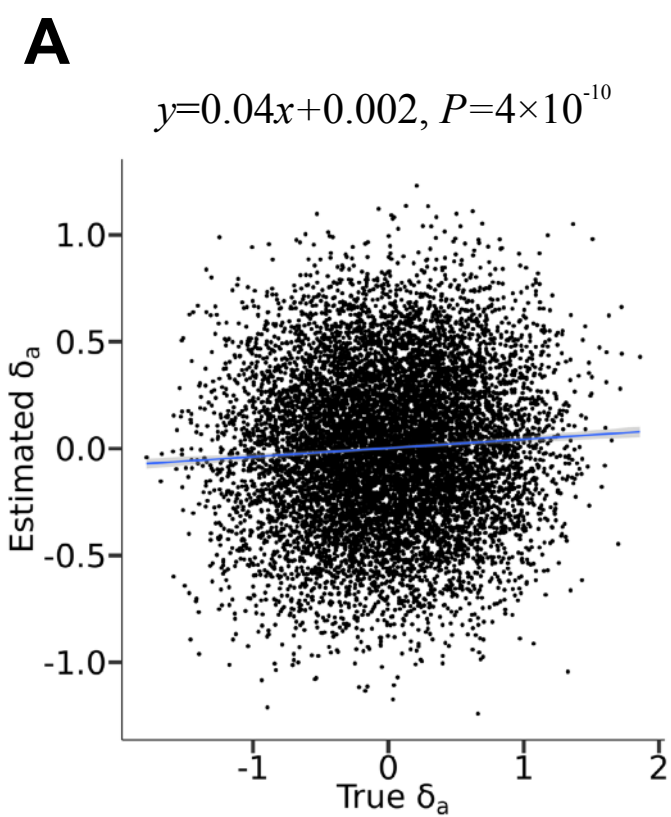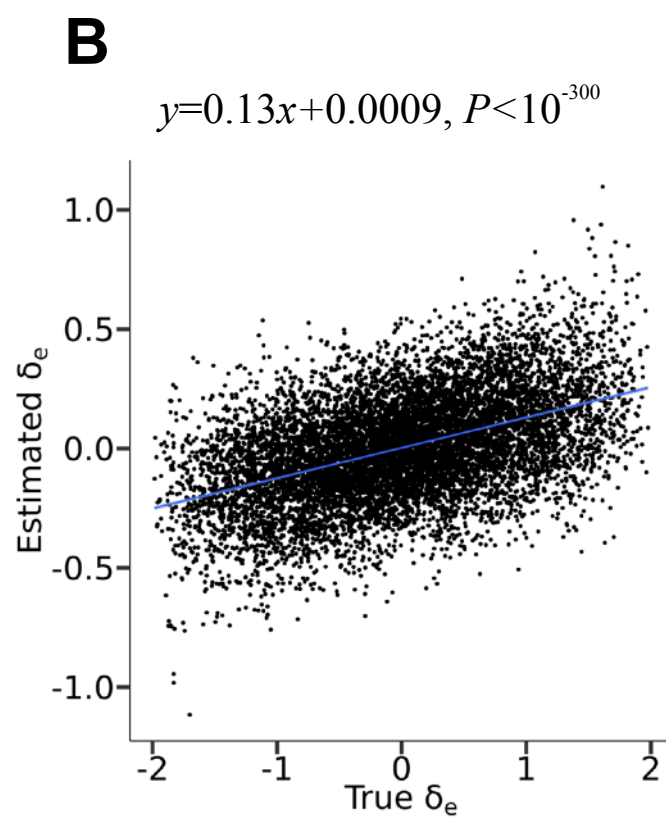

**Figure S7**

Supplement: S7 Fig — (A) The magnitude of δa estimated from allele-specific ATAC-seq is much smaller than the true δa. (B) The magnitude of δe estimated from allele-specific single-cell RNA-seq is much smaller than the true δe. (PDF) [file pgen.1008389.s007.pdf]
