# Supplementary material for: Chromosome-wide co-fluctuation of stochastic gene expression in mammalian cells
Source: PLoS Genet. 2019 Sep 16;15(9):e1008389. doi: 10.1371/journal.pgen.1008389 (PMC6762216; doi:10.1371/journal.pgen.1008389)
Supplement: Attachment — Submitted filename: Response.pdf [file pgen.1008389.s008.pdf]

Response to the reviewers

We thank the three reviewers for their valuable comments, which have helped improve our manuscript. Below please find our point-to-point response in blue.

Reviewer #1:

The study of Sun & Zhang reveals an interesting coupling between gene positioning along a chromosome and fluctuations, indicating that cis-correlations are higher than trans-correlations in the context of homologous chromosomes. The majority of tests are convincing, in particular the one showing that the effect becomes more prominent when they focus on highly expressed genes. The fact that the authors study the differential behavior of linked genes in comparison to their homologous counterparts (within a certain cell type) is clear in some but not in other contexts. Therefore, I suggest addressing the points below before the publication of the manuscript.

We thank the reviewer for the overall positive evaluation.

Major remarks
1) The comparison of expression fluctuations with chromosomal looping and chromatin accessibility clearly follows physical models of gene expression. However, the hypothesized link between expression fluctuations and protein complexes " the expression co-fluctuation of genes encoding components of the same protein complex is likely advantageous." should be examined in more detail. The authors implicitly assume the formation of protein complexes is enhanced when the subunits expressed in a coordinated fashion, irrespective of the basis of comparison. However, it is possible that there is no difference in the correlation with homologous and heterologous interchromosomal fluctuations. This is important since each subunit of the dimer is expressed from a maternal and paternal chromosome even if they are positioned on homologous and heterologous chromosomes. Therefore, either the simulations should include different scenarios of chromosomal fluctuations or the above conclusion should be explained statistically more rigorously.

Because two of the four allele pairs between two linked genes in a diploid are unlinked, the reviewer wonders whether linkage increases the mean concentration of a protein complex in a diploid. As described on page 8 of the main text and shown in Fig. S3, the correlation between the total expression of gene A and that of gene B is higher for linked than unlinked genes in diploid cells. Therefore, our conclusion holds in diploids. The simulation in Fig. 4A considers protein concentrations, so it directly applies to both haploid and diploid cases. The only difference between them is that $[A]_t$ and $[B]_t$ tend to have smaller CV in diploids than haploids. We have added the above points to page 16 of the main text where Fig. 4A is explained.

Minor remarks:
2) A. The authors compare stochastic intra-chromosomal and inter-chromosomal correlations. This is important because most studies (as mentioned in the discussion) do not distinguish intra-chromosomal effect. This is mentioned at several places, notably in the abstract, in the last paragraph of the introduction , first section of the results,…. However, the authors should not

assume that the general audience understands the term "linked" and should explain that in more general terms in the author summary. For example, the authors could explain in the author summary that each individual receives a maternal and paternal copy of the same chromosome.
B. Figure 1 or Figure 2A should include the full depiction of pair(s) of homologous chromosomes so that it becomes clear what the authors mean.

Following the suggestion, we have rewritten the author summary to explain the maternal and paternal chromosomes, etc. In addition, we added such information to Fig. 2A legend.

3) The interchromosomal fluctuations may have a substantially stronger effect on gene choice than on protein complexes. Therefore, I suggest the authors discussing stochastic gene choice in the context of their results. The Protocadhering gene array is prototypical example where stochastic gene expression is very strongly depends on intrachromosomal looping controlled by the binding of the looping factor CTCF in neuronal progenitors (PMID: 15640798, 30257211). Therefore, the intrachromosomal fluctuations and conformational changes may have a strong impact on the choice and number of expressed protein isoforms of this membrane protein, which can underscore the biological relevance of the fluctuations studied by the authors.

We agree. Gene array is a set of genes that are closely linked on the same chromosome. Specific mechanisms are required to selectively express a subset of genes from this large group. Some of these mechanisms require physical proximity. Therefore, the linkage effect studied here might have important implications beyond gene expression co-fluctuation. We have added the above points and cited the relevant papers suggested by the reviewer on page 23.

Reviewer #2:

In this study, the authors investigated co-fluctuation of chromosomally-linked genes. This phenomenon was first discovered experimentally by a previous study using fluorescent reporters (Raj et al, 2006). Here the authors analyzed the existing single-cell RNA seq data and showed that this could be a genome-wide phenomenon. To this end, they showed statistically the expression correlations between linked alleles (on the same chromosome) of two genes are greater than those between unlinked alleles. With the support of additional analysis, they further demonstrated that the 3D proximity and chromatin co-accessibility could be the underlying mechanism of this effect. Finally, they proposed that, through natural selection, genes encoding components of the same complexes might be organized onto the same chromosomes to correlate their expression.

I find this paper well-written and scientifically interesting. Gene expression coordination in single cells has been an important topic of research. The authors performed a careful analysis of existing data and raised an intriguing model. I would recommend the acceptance of this manuscript for publication shall the concerns below be addressed.

We thank the reviewer for the overall positive evaluation.

1. I would think that the most direct way to coordinate gene expression of functionally-related

genes in individual cells is through sharing the same trans or cis-regulatory factors. Is there a way to quantitively compare the contribution of this mechanism with the contribution of chromosomal linkage? I suspect that the former one might play a major role. The paper is interesting either way, but a careful discussion and comparison will be helpful.

This is a good question. But it is difficult to compare the relative contributions of regulatory element sharing and physical linkage to the expression co-fluctuation of genes, because the extent to which two genes share regulatory elements is generally unknown. However, in one extreme case, the two effects can be compared. This extreme case is the consideration of expression co-fluctuation of two alleles of the same gene, because the two alleles could share all regulatory elements. In this extreme case, the contribution of the linkage effect is estimated to be one thirteenth the contribution of sharing regulatory elements. We added this comparison to the main text (pages 19-20) and our method of comparison to Materials and Methods (page 30).

2. Related to point 1, for natural selection, would sharing regulatory elements provide more plasticity than reorganizing the genome? Again, a careful discussion would be needed. Genome re-organization does not seem like the more intuitive way to ensure gene expression coordination.

Although sharing regulatory elements may be a more effective mechanism to control gene expression co-fluctuation, linkage is an additional mechanism that can further improve co-fluctuation. This can be seen by comparing the scenario of two genes with identical regulatory sequences but are located on different chromosomes with the scenario of two genes with identical sequences and are located on the same chromosome (see response to the previous comment). This additional improvement in co-fluctuation can be important under certain circumstances, as shown recently for genes encoding enzymes of the yeast galactose use pathway [1]. This discussion is added to page 20.

3. In addition to genes encoding components of the same complexes, I wonder whether the same organization rule can be applied to the genes encoding components of the same signaling or metabolic pathways? If not, why?

Protein complexes are by no means the only group of genes for which co-fluctuation can be advantageous. Previous work suggested that co-fluctuation can be beneficial for genes on the same signaling or metabolic pathway [2, 3]. We have added the discussion and citations in our manuscript (page 22).

<u>Reviewer #3:</u>

The manuscript from Mengyi Sun and Jianzhi Zhang lays out the novel and provocative idea that coordinated fluctuations in gene expression stem from fluctuations in local histone H1 concentrations.

In support of this idea, they show that expression of "linked" (immediate nearest neighbors along the same chromosome) genes is more correlated than expression of the same pair of genes across

homologous chromosomes. Suprisingly, this is true even when the two genes are seperated by over 100Mb, suggesting a 3D, not 1D, cause. Consistent with this, but unsuprisingly, alleles on the same chrommosome are more frequently in physical contact with each other than alleles on homologous chromosomes.

As a mechanism they propose local diffusion of H1, and in support of this mechanism they find that protein complex members whose expression is negativly correlated with H1 mRNA levels are more likely to be linked.

The ideas are provocative and, while, as the authors point out, the effect sizes are weak, some of this may be due to imperfect matches between experimental data and experimental error. In addition, evolution can act on weak effect sizes.

We thank the reviewer for the overall positive evaluation.

However, their model makes a major prediction that I'm suprised they didn't test, and I would like to see tested before the paper is published. The authors propose that 1D distance doesn't matter; only 3D distance. This predicts that the correlation shown in Fig1D should be even stronger if 3D distance is plotted on the X axis. Furthremore, being nearest neighbors (linked) shouldn't be the most predictive feature. HiC contact frequency should be. If you recalculate something like Figure 1B but bin into multiple groups, binning genes by contact frequency and/or distance in 3D space, instead of only into linked vs unlinked, does the effect still hold? In other words, genes that are unlinked, but still close in 3D space, should exhibit equally strong co-expression.

Fig. 3H shows the correlation between 3D distance and expression co-fluctuation. Note that although the impact of 3D distance (Fig. 3H) appears weaker than the impact of genomic distance (Fig. 2D) on δ_e, these two plots are not directly comparable because of the following reasons. First, the Hi-C contact frequency is not an accurate measure of 3D proximity, especially for region pairs that rarely contact, which apply to the vast majority of region pairs (Fig. 3C). By contrast, genomic distance is measured accurately. Second, the resolution of the Hi-C data used is much lower (500 kb) than that of the genomic distance (1 bp). Third, the Hi-C data and gene expression co-fluctuation data are not from the same cell type, which reduces the observed impact of 3D distance. The above points have been added to page 13.

Minor Point:
"While each of the four correlations could be positive or negative, in the large data analyzed below, they are mostly positive and show approximately normal distributions across gene pairs examined."

I'd like to see the histograms of these four correlations (re(A1,B1), re(A2,B2), etc)

We plotted the distributions in Fig. S1. Note that the distribution of re(A1, B2) cannot be distinguished from that of re(A2, B1), so they are merged.

"All statistical analyses were performed using custom R and python scripts that are available

upon request." It's 2019; this should no longer be acceptable. Please put all code and intermediate (processed) data on github or some other repository. The PLoS Genetics data availibility guidelines state that, if the data are not made publically availible, "The reasons for restrictions on public data deposition must also be specified. "

As now described in the manuscript, we have uploaded codes for all figures and uploaded all meta-data necessary for the statistical tests to GitHub and Mendeley, respectively.

References:
[1] Xu, H. et al. (2019) Synchronization of stochastic expressions drives the clustering of functionally related genes. Science Advances, in press.

[2] Veitia, R.A. (2004) Gene dosage balance in cellular pathways: implications for dominance and gene duplicability. Genetics 168 (1), 569-574.

[3] McGary, K.L. et al. (2013) Physical linkage of metabolic genes in fungi is an adaptation against the accumulation of toxic intermediate compounds. Proceedings of the National Academy of Sciences USA 110 (28), 11481-11486.

Response to *PLOS Genetics* editorial staff on "data not shown"

In the original manuscript, we wrote on page 36 that "The blue line shows the linear regression of the binned data. For better viewing, one bin (X=156, Y= -0.02) is not shown in the figure; the extreme δ_a of the bin is probably due to the small sample size of the bin (n = 13)." *PLOS Genetics* editorial staff informed us that "data not shown" is not allowed in the journal. This is actually not a typical "data not shown" case, because by providing in this sentence the information for the bin not shown on the figure, we actually provided all data. To avoid confusion, we have replaced "not shown" with "is omitted".
